# Evaluating the impact of homoeopathy in managing the morbidity associated with grade III and IV lower limb lymphoedema in adults with lymphatic filariasis

**DOI:** 10.3389/fpara.2026.1939673

**Published:** 2026-10-22

**Authors:** Panaparambil Azis Suhana, Jain Vij Shruti, Sujata Choudhury, Jaya Gupta, Vasudeva Guddattu, Prakash Kumar Sahoo, Charu Sehgal, Preeti Bhandari, Monalisha Das, Prasad Varamballi, Subhash Kaushik

**Affiliations:** 1 Central Council for Research in Homoeopathy, Ministry of Ayush, Government of India, New Delhi, India; 2 National Institute of Homoeopathy, Ministry of Ayush, Government of India, New Delhi, India; 3 Regional Research Institute for Homoeopathy, Puri, Odisha, India; 4 Drug Proving Unit (Homoeopathy), Bhubaneswar, Odisha, India; 5 Department of Applied Statistics and Data Science, Prasanna School of Public Health, Manipal Academy of Higher Education, Manipal, Karnataka, India; 6 ICMR-Regional Medical Research Centre, Bhubaneswar, Department of Health Research, Ministry of Health & Family Welfare, Government of India, Odisha, India; 7 Manipal Institute of Virology, Manipal Academy of Higher Education, Manipal, Karnataka, India

**Keywords:** dermatology life quality index, homoeopathy, lymphatic filariasis, lymphoedema, water displacement

## Abstract

**Introduction:**

Lymphatic filariasis is a major public health burden, particularly in endemic regions, with millions suffering from chronic lymphoedema and hydrocele. While mass drug administration aims to restrict transmission, effective morbidity management remains a significant challenge. Homoeopathy has reported potential in managing lymphoedema, yet evidence in cases of grade III and IV lymphoedema is limited.

**Methods:**

This open-label, exploratory, interventional study, conducted at the Regional Research Institute for Homoeopathy, Puri, Odisha, India, aimed to evaluate the effects of individualized homoeopathic medicines in managing grade III/IV unilateral/bilateral lower-limb lymphoedema associated with lymphatic filariasis. Outcomes included changes in limb circumference (Australasian Lymphology Association standards), limb volume (Water Displacement Method), health-related quality of life (7D5L scale), dermatological changes (Dermatology Life Quality Index), acute adenolymphangitis episodes, skin health, and serological markers over one year. A total of 104 eligible participants were enrolled, and followed-up monthly for one year. 61 participants dropped out of the study, and 43 participants successfully completed all the follow-ups.

**Results:**

Statistically significant improvements were observed in quality of life and dermatological indices (p-value < 0.0001), along with alleviation of limb volume. Decreased Og4C3 and TNF-α levels, resolution of entry lesions and ulcers, and reduction in occurrence of fungal infections were documented.

**Discussion:**

The study demonstrated the potential benefits of homoeopathy in managing advanced filarial lymphoedema by improving quality of life. Further controlled studies with larger sample are warranted.

**Clinical trial registration:**

https://ctri.nic.in/Clinicaltrials/pmaindet2.php?EncHid=MjY2NzE=&Enc=&userName=, identifier CTRI/2018/06/014591.

## Graphical Abstract

**Figure:**
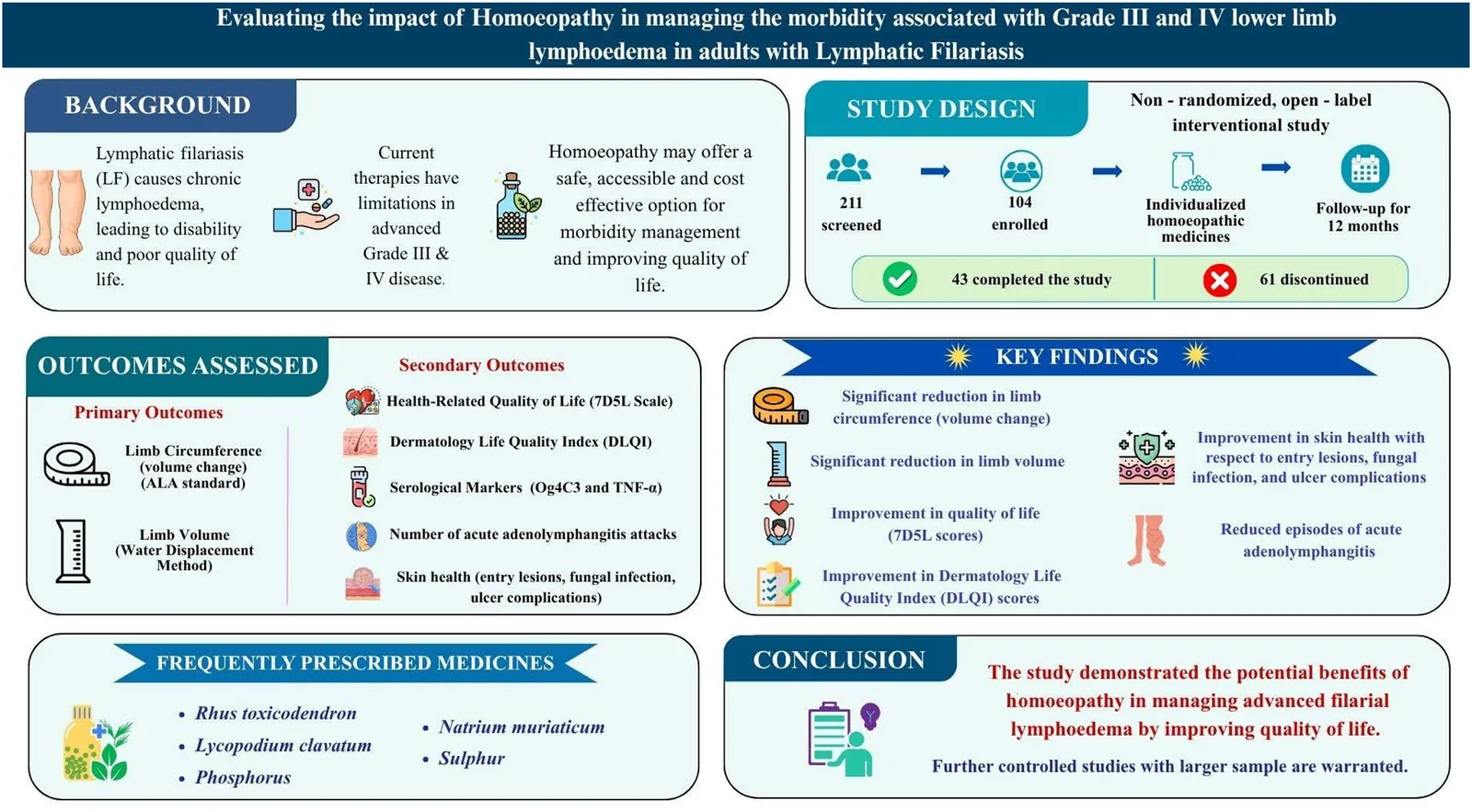

## 1 Introduction

Lymphatic filariasis (LF), commonly known as elephantiasis, is a vector-borne neglected tropical disease caused by parasitic nematodes, namely *Wuchereria bancrofti, Brugia malayi*, and *Brugia timori*. Humans serve as the primary definitive hosts and transmission occurs through infected mosquitoes, predominantly of the *Culex*, *Anopheles*, and *Aedes* species. Mosquitoes ingest microfilariae from infected individuals during a blood meal, which subsequently develop into infective third-stage larvae within the vector. These larvae are transmitted to humans during subsequent bites, migrate to the lymphatic vessels, mature into adult worms, and produce microfilariae that circulate in the bloodstream, thereby sustaining transmission. This ultimately leads to progressive lymphatic dysfunction, chronic inflammation and structural damage, resulting in lymphoedema (World Health Organization (WHO), 2024; Goldin and Juergens, 2026).

Although considerable progress has been achieved in the control of LF, the disease continues to pose a substantial challenge to public health in many endemic regions. The World Health Organization 2024 estimates indicate that the global burden of LF declined substantially between 2000 and 2018, with the number of affected individuals falling to approximately 51 million, reflecting a reduction of 74% since the establishment of the Global Programme to Eliminate Lymphatic Filariasis in 2000. However, the disease remains responsible for a significant burden of chronic morbidity. Nearly 36 million individuals suffer from long-term complications, including hydrocoele in about 25 million men and lymphoedema in around 15 million people. Moreover, approximately 657 million individuals across 39 countries continue to face the risk of infection (World Health Organization (WHO), 2024). As a neglected tropical disease associated with disability, stigma and reduced quality of life, LF has been identified as a priority for elimination, with a global target year of 2030. The progress towards this goal is evident, with 21 countries having achieved elimination by 2024 (Jabir and Rahi, 2025; World Health Organization (WHO), 2026).

Globally, the WHO South-East Asian Region bears the largest proportion of the population at risk of LF. Owing to its vast susceptible population and considerable disease burden, India occupies a central position in achieving global elimination goals, accounting for nearly 62% of the worldwide at-risk population. The disease remains endemic in 348 districts across 20 states and union territories, with nearly 0.62 million individuals affected by lymphoedema and approximately 405 million people vulnerable to infection (Jabir and Rahi, 2025). In response to this persistent burden, the Government of India initiated the National Programme to Eliminate Lymphatic Filariasis in 2004, originally aiming for elimination by 2020, a target later revised to 2027 (Tripathi et al., 2022). The programme follows an integrated five-pronged strategy encompassing mass drug administration (MDA), morbidity management and disability prevention, integrated vector control, intersectoral coordination, and the application of digital technologies to strengthen disease surveillance and control measures (National Center for Vector Borne Diseases Control (NCVBDC), 2024).

Although MDA has significantly contributed to reducing disease transmission, advancements in morbidity management and disability prevention have remained relatively limited. In particular, advanced lymphoedema continues to pose a major therapeutic challenge because of irreversible lymphatic impairment, poor responsiveness to conventional pharmacological treatment, and the scarcity of effective therapeutic interventions. At present, management of chronic lymphoedema primarily depends on non-pharmacological approaches such as limb hygiene, skin care, physical exercise, and compression therapy. However, adherence to these interventions is often inadequate, and treatment outcomes in advanced stages remain unsatisfactory. In addition, restricted access to specialized healthcare services, insufficient awareness regarding self-care and limb hygiene practices, and the enduring social stigma associated with the disease collectively impede effective long-term management (Medeiros et al., 2022).

Advanced LF-associated lymphoedema, particularly Grades III and IV, represents the most severe manifestation of the disease and is characterized by irreversible limb swelling, dermal thickening, fibrosis, and recurrent episodes of acute dermatolymphangioadenitis. These pathological alterations result from prolonged lymphatic dysfunction and chronic inflammatory processes, leading to pain, impaired mobility, and substantial functional disability. Repeated secondary bacterial infections further exacerbate lymphatic injury, perpetuating inflammation and fibrosis and thereby accelerating disease progression. Beyond the physical consequences, affected individuals frequently experience profound psychosocial difficulties, including social exclusion, emotional distress, depression, and economic hardship, all of which markedly diminish their overall quality of life (World Health Organization (WHO), 2013; Vasconez-Gonzalez et al., 2025).

Clinically, LF presents a wide spectrum ranging from acute adenolymphangitis, along with fever and other systemic symptoms in early stages to chronic irreversible conditions, such as lymphoedema and hydrocele. The microfilariae carriers are typically asymptomatic, contributing silently to disease transmission. The progression to chronic disease is believed to involve complex interactions between host immune-mediated inflammatory responses, secondary bacterial infections, and genetic predisposition. Tumor necrosis factor-alpha (TNF-α) plays an important role in disease pathogenesis by regulating inflammation through its receptors TNFR-I and TNFR-II; with alterations in TNFR-II expression and polymorphisms being associated with fibrosis and lymphatic obstruction (Panda et al., 2011).

Accurate diagnosis of LF is essential for effective management and control. There are two highly sensitive and specific tests available for detecting the circulating filarial antigens in bancroftian filariasis that can identify amicrofilaremic and microfilaremic infections, namely, Og4C3 ELISA and Filaria Test Strip (FTS). The Og4C3 ELISA test employs an anti-Onchocerca antibody, specifically the Og4C3 monoclonal IgM antibody, which is derived from the bovine filarial parasite, *Onchocerca gibsoni*. While the FTS lateral rapid test utilizes AD12 monoclonal antibody, derived from the canine filarial parasite, *Dirofilaria immitis* (Rahumatullah et al., 2019).

The current pharmacological interventions, including diethylcarbamazine, ivermectin, and albendazole, administered as a part of triple drug regimen in MDA programmes, primarily target microfilariae and help interrupt transmission. However, their limited efficacy against adult worms restricts their therapeutic benefit in the affected individuals (Fordjour et al., 2023). Consequently, there is no definitive curative treatment for LF, and management primarily focuses on preventing painful acute inflammatory episodes and slowing disease progression (Kakakhel et al., 2025). Although doxycycline has demonstrated some benefit in early-stage lymphoedema (Grades I and II), its effectiveness in advanced stages remains uncertain, underscoring a significant therapeutic gap (Medeiros et al., 2022). For patients suffering from advanced lymphoedema, this therapeutic gap underscores the need for approaches that can contribute not only in managing complications associated with the disease but also in preserving mobility, functional independence, skin health, and overall quality of life.

In this context, Homoeopathy has emerged as a potential adjunctive approach in the management of lymphoedema associated with LF. A study conducted by the Central Council for Research in Homoeopathy (CCRH) suggests that homoeopathic treatment is as effective as conventional medicine in managing acute adenolymphangitis associated with LF (Gupta et al., 2018). Additional studies have also reported encouraging outcomes in managing early-stage lymphoedema (Grades I and II), and in reducing the frequency of acute episodes (Subramanyam et al., 1990; Rastogi and Mishra, 1991; Kumar and Mishra, 1994; Prusty et al., 2025). However, evidences evaluating the effectiveness of homoeopathic medicines in advanced lymphoedema (Grades III and IV) remain scarce. To the best of current knowledge, there is a lack of longitudinal studies systematically assessing therapeutic outcomes in advanced LF using objective clinical, serological, and quality of life measures.

Therefore, the present study was undertaken to address this gap by investigating the effect of homoeopathic medicines in the management of Grade III and Grade IV lower limb lymphoedema resulting from LF over a period of 01 year. The assessment included measurement of limb circumferences using standardized tape measurement techniques in accordance with the Australasian Lymphology Association (ALA) standards, along with estimation of limb volume using the Water Displacement Method. In addition, health-related quality of life was evaluated using 7D5L scale, and dermatological outcomes through the Dermatology Life Quality Index (DLQI). The frequency of acute adenolymphangitis episodes was also recorded to assess disease activity. Serological parameters, including Og4C3 circulating filarial antigen and TNF-α levels were also measured. Furthermore, skin health was assessed before and after treatment, focusing on skin integrity, entry lesions, fungal infections, and ulcer-related complications.

## 2 Methods

### 2.1 Study design and setting

This open-label, exploratory, interventional study without a parallel control group was carried out at the Outpatient Department of the Regional Research Institute for Homoeopathy, Puri, from October 2018 to October 2023. The individualized homoeopathic medicines were prescribed to the study participants in centesimal potencies in accordance with the homoeopathic principles for a duration of 01 year, with monthly follow-up visits. The study was prospectively registered in the Clinical Trials Registry- India (CTRI/2018/06/014591 dated 20.06.2018).

### 2.2 Participants

Both male and female participants aged 18–99 years with a confirmed clinical history of LF, and presenting with Grade III/IV unilateral or bilateral lymphoedema accompanied by characteristic dermatological presentations, with willingness to give written consent, were enrolled in the study. Participants with controlled diabetes mellitus and hypertension were also considered eligible. Those presenting with acute adenolymphangitis associated with Grade I/II lymphoedema, and non-filarial lymphoedema (congenital, deep vein thrombosis, malignancies, irradiation, surgery, etc.) were excluded from the study. Individuals with a history of life-threatening systemic illnesses (tuberculosis, cancer, acquired immunodeficiency syndrome, cardiovascular disorders, etc.) and those receiving lifelong medication for chronic diseases were not enrolled in the study. Pregnant or lactating women and participants assessed as unfit by the Site Investigator were also excluded.

Participants underwent a two-stage screening procedure involving a preliminary screening by the OPD physician and the subsequent eligibility assessment by the study site investigator. Following this, the eligible participants were enrolled after obtaining written informed consent. A comprehensive case history of each participant was recorded using a structured case-taking proforma. Personalized symptom totality was constructed for each participant from common and characteristic symptoms recorded during case-taking and the symptom analysis was done using Radar Opus 2.0 licensed software to derive indicated medicines. Further, the homoeopathic medicines procured from Good Manufacturing Practices (GMP) certified pharmaceutical manufacturers were prescribed to the participants orally in centesimal potencies, that is, 30C, 200C, 1M and 10M, as per the requirement of individual cases. The selection of medicine, potency, dosage, and frequency of repetition were individualized according to the clinical presentation and symptom response of each participant, in accordance with the principles of Homoeopathy laid down in the Organon of Medicine (Hahnemann, 2011). As the intervention was individualized, a uniform fixed dose, potency, or repetition schedule was not applied to all participants.

The participants were followed-up monthly for duration of 01 year. At each follow-up visit, the clinical response and changes in the presenting symptomatology were assessed to determine whether repetition of the indicated medicine was required. When clinical improvement was observed, the previously prescribed medicine was not repeated, and the identical non-medicated globules were administered during the follow up visits. When no change was noted, the indicated medicine was repeated at the same dose; and subsequently in higher potencies, if necessary, based on the individual clinical response. Thus, repetition of the medicine was response-dependent rather than being administered routinely at every monthly follow-up. Participants not responding to treatment were referred to the General OPD for further management. Separate fields were included in the case recording format to document any adverse events during each follow-up visit.

### 2.3 Variables

The primary outcome of the study was to evaluate and compare the clinical improvement in Grade III/IV unilateral or bilateral lower limb lymphoedema after 01 year of homoeopathic intervention. This was determined by measuring the changes in limb circumference using a tape measure in accordance with the ALA standards, along with assessment of limb volume using the Water Displacement Method.

The secondary outcome measures included assessment of health-related quality of life using the 7D5L scale, estimation of serological markers (Og4C3 and TNF-α), evaluation of dermatological changes using DLQI. Additionally, the frequency of acute adenolymphangitis attacks, and overall skin health with respect to entry lesions, fungal infection, and ulcer complications were evaluated after one year of treatment.

### 2.4 Data sources/measurement

Baseline data were collected from study participants during their initial visit to the Outpatient Department. At baseline, participants underwent enrolment, intervention initiation, and assessment of limb circumference, water volume, 7D5L scores, DLQI scores, serological parameters, acute adenolymphangitis episodes, and skin health. Limb circumference, water volume, acute adenolymphangitis episodes, and skin health were assessed at all the follow-up visits, while 7D5L and DLQI scores were evaluated at 3^rd^, 6^th^, 9^th^ and 12^th^ month follow-ups, with serological parameters reassessed at the final follow-up visit. The participant timeline and specific assessment intervals are outlined in **Table 1**.

**Table 1:** Participant timeline and assessment intervals.

Procedures	Baseline	FU 1	FU 2	FU 3	FU 4	FU 5	FU 6	FU 7	FU 8	FU 9	FU 10	FU 11	FU 12
Consent	✔												
Enrolment	✔												
Intervention	✔	✔	✔	✔	✔	✔	✔	✔	✔	✔	✔	✔	✔
Follow-up		✔	✔	✔	✔	✔	✔	✔	✔	✔	✔	✔	✔
Limb circumference measurement	✔	✔	✔	✔	✔	✔	✔	✔	✔	✔	✔	✔	✔
Water volume	✔	✔	✔	✔	✔	✔	✔	✔	✔	✔	✔	✔	✔
7D5L scores	✔			✔			✔			✔			✔
Serological estimation of Og4C3 and TNF-α	✔												✔
DLQI scores	✔			✔			✔			✔			✔
Number of acute adenolymphangitis attacks	✔	✔	✔	✔	✔	✔	✔	✔	✔	✔	✔	✔	✔
Skin health (entry lesions, fungal infection, ulcer complications)	✔	✔	✔	✔	✔	✔	✔	✔	✔	✔	✔	✔	✔

FU, Follow-up; DLQI, Dermatology Life Quality Index; TNF-α, Tumor necrosis factor- alpha.

#### 2.4.1 Limb circumference measurement

In accordance with the ALA standards, the participant was instructed to lie supine, with the leg in slight abduction on a measuring board, keeping the foot flat and the toes pointed upwards, while the opposite leg was adequately supported to prevent pelvic rotation. The midpoints of the medial and lateral malleoli were identified to ensure correct alignment of the leg and consistent repositioning for subsequent follow-ups. Additionally, the medial and lateral aspects of the limb were marked at the central points of the metatarsophalangeal and tarsometatarsal joints, as well as at 10 cm intervals beginning from the plantar surface of the foot and extending proximally up to 60 cm along the thigh, adjusted according to the height of the participant (Australasian Lymphology Association, 2004).

A tape measure was used to record the circumferences of each limb segment. The volume of each segment was subsequently computed using the truncated cone formula*, V = h/12π (C_1_^2^+ C_1_C_2_+ C_2_^2^)*, where *V* signifies the volume, *h* corresponds to the length between proximal and distal circumference points, *C_1_* refers to proximal and *C_2_* denotes distal circumferences. The total limb volume was calculated by adding the volumes of each segment. The absolute change in volume was then determined for each subsequent assessment relative to baseline (Australasian Lymphology Association, 2012; Farina et al., 2024).

#### 2.4.2 Water volume

The limb (water) volume was measured using the Water Displacement Method, based on Archimedes’ principle. The whole limb was submerged vertically in a brim-filled container, with the participant standing on the contralateral limb positioned on an adjustable platform placed next to it. The displaced overflown water representing the volume of the immersed limb, was collected and measured by determining its weight in a calibrated vessel (Rabe et al., 2010).

#### 2.4.3 7D5L scale

7D5L scale is an assessment scale employed to evaluate the health status of individuals with LF in seven domains and five levels. The tool measures the impact of LF on physical health (mobility, self-care, usual activity, and pain/discomfort); psychological health (anxiety/depression, and cognition); and social participation. *Mobility* was evaluated based on the ability of the participants to perform tasks such as walking, moving about, travelling, and driving. *Self-care* was assessed addressing the daily activities like eating, bathing, dressing, and toileting. *Usual activity* pertaining questions included tasks referring to professional or household work such as cooking, shopping, caregiving, painting, etc. *Pain/discomfort* assessment covered both localized and generalized discomfort. The psychological health, concerning *anxiety/depression* was evaluated based on the self-reported depression and anxiety symptoms, including sadness, nervousness, low self-esteem, and loss of interest. *Cognition* was also assessed which involved ability to learn, memorize, concentrate, understand, and complete tasks. *Social participation* was evaluated by examining the participant’s involvement in social interactions, such as maintaining relationships, engaging with family and friends, and attending social gatherings. Each domain was scored on a 5-point rating scale ranging from 0 to 4, corresponding to no problem (0), mild (1), moderate (2), severe (3), and extreme problem (4). The overall severity score was obtained by adding the domain scores, yielding a possible range from 0 to 28 (Harichandrakumar et al., 2006).

#### 2.4.4 Dermatology Life Quality Index (DLQI)

DLQI, introduced by AY Finlay and GK Khan in 1992, is a self-administered, patient-reported instrument designed to ascertain the effect of dermatological problems on health-related quality of life. The questionnaire consists of 10 items covering six domains of symptoms and feelings, daily tasks, leisure pursuits, occupational and academic activities, interpersonal relationships, and treatment effects. Each item is rated on a 4-point scale from 0 (not affected) to 3 (very much affected). The overall mean score is computed by adding all the domain scores ranging between 0 and 30. The scores are categorized as 0-1 (no effect), 2-5 (small effect), 6-10 (moderate effect), 11-20 (very large effect), and 21-30 (extremely large effect). Higher scores reflect greater impairment in quality of life (Mohammed et al., 2005). A free license was secured from Cardiff University to use this questionnaire for this study.

#### 2.4.5 Serological estimation

The blood samples were collected from the study participants at baseline and 12^th^ follow-up. The following assays were employed for the estimation of serological markers, namely, Og4C3 and TNF-α:

##### Og4C3 antigen assay

A 2–3 ml blood sample was drawn from each participant by a skilled technician under sterile conditions. After allowing blood to clot, it was centrifuged at 2,000 x g for 10 minutes to separate the serum. The *Wuchereria bancrofti* circulating filarial antigen was detected using the TropBio Filariasis Antigen Og4C3 ELISA kit. To prepare heat-stable antigen, serum was boiled with diluent at 100°C, then centrifuged, and the resultant supernatant was used in the assay. Standards, controls, and test samples were dispensed into pre-coated wells and incubated at 37 °C for 60 minutes. After three washes, the wells were treated with anti-Onchocerca antibody for 45 minutes, followed by another wash, and incubation with HRP-conjugated secondary antibody for 45 minutes. Following another wash, substrate was added and incubated in dark for 15 minutes. The reaction was then terminated and absorbance was read at 450 nm using an ELISA reader. All incubations occurred in a humid chamber with triple washing at each step. The antigen concentration was determined using the standard calibration curve.

##### TNF-α assay

The assay was carried out using the *R&D SYSTEM Duo Set ELISA Human TNF-α* kit, a standard sandwich ELISA designed for quantification of natural and recombinant human Tumor Necrosis Factor-alpha. A 96-well microplate was coated with 100 µL of diluted capture antibody and kept overnight at room temperature. After washing with wash buffer, the well was blocked with 300 µL of blocking buffer for 1 hour. Following another wash, 100 µL of standards or samples was dispensed into the wells and incubated for 2 hours. Subsequently, detection antibody and streptavidin-HRP were added, with respective incubation periods of 2 hours and 20 minutes, with washing carried out after every incubation. The substrate solution was further added and allowed to react in the dark for 20 minutes and the reaction was terminated by adding 50 µL of stop solution. Optical density was then measured at 450 nm using an automated ELISA microplate reader (Erba Lisa Scan^®^ EM). The concentration of TNF-α was determined based on the standard calibration curve.

### 2.5 Bias

Although the study design was methodologically robust, potential biases may still exist. Random sampling of the study participants was done to minimize selection bias. The use of validated and standardized questionnaires reduced measurement inaccuracies and information bias. Accurate records were maintained to identify potential biases and errors. Missing data were handled using K-Nearest Neighbors (KNN) imputation method, thereby reducing the risk of attrition bias.

### 2.6 Sample size

Given the limited prior evidence on the expected magnitude of improvement in morbidity indicators, a conservative approach was adopted, assuming that 50% of the study population would improve in clinical outcomes during follow-up. Assuming 95% confidence level and an allowable error margin of 10%, the sample size was calculated using the formula:


$$N=\frac{{\left(Z\right)}^{2}\times p(1-p)}{e^{2}}$$

where Z is the Z-score for the confidence level (1.96 for 95% confidence), p = the expected proportion (0.5), and e = the allowable error (0.10).


$$N=\frac{{\left(1.96\right)}^{2}\times (0.5)(0.5)}{{\left(0.10\right)}^{2}}=96$$

Thus, the minimum required sample size was approximately 96 participants. Considering a potential 20% attrition during the follow-up, the target sample size was rounded to 100 participants. The subsequently longitudinal analyses used repeated-measures ANOVA and Friedman test across multiple follow-up time points. The initial proportion-based sample size calculation did not directly account for within-subject correlation, the number of repeated measurements, or the expected effect size for longitudinal changes.

### 2.7 Quantitative variables

Since the data recorded for limb circumference were not normally distributed, absolute change in limb volume was expressed as Median ± Interquartile Range (IQR). The Friedman test was applied to assess the temporal variations separately for both the limbs. However, the water volume data were normally distributed, hence, were presented as Mean ± Standard Deviation (SD) at each time point. Repeated-measures ANOVA was applied to evaluate temporal trends in water volume. Thereafter, Tukey’s *post hoc* multiple comparison test was applied for pairwise comparisons after observing overall statistical significance. The secondary outcome measures, namely, DLQI and 75DL scores, were also summarized as Mean ± SD, and analyzed over time with repeated-measures ANOVA.

### 2.8 Statistical methods

The study datasets included baseline anthropometric variables, including height, weight, and body mass index, along with detailed circumferential measurements of both legs at various anatomical landmarks and parameters concerning foot swelling. All statistical analyses were performed with GraphPad Prism (version 9.0). Shapiro-Wilk test was used to test for normality in each sample set. KNN imputation was applied to address the missing values in the primary outcome measures. The proportion of missing data was 20.2%, and imputation was performed using k=5. KNN imputation was selected because the analysis was based on data from 43 study participants, with missing observations occurring across multiple follow-up time points. KNN imputation allows missing values to be estimated using information from participants with similar observed characteristics, thereby retaining participants with partially missing data and avoiding the substantial loss of observations that would result from a complete-case analysis.

The resulting imputed dataset was used for statistical analysis. Given that the analysis included 43 study participants who have completed the follow-ups and involved the multiple follow-up time points, a complete-case analysis would have resulted in substantial loss of observations and reduced statistical power. A complete-case sensitivity analysis was therefore not performed. The Friedman test was applied to assess change in limb circumferential measurements over time, while repeated-measures ANOVA was used for water volume, DLQI, and 7D5L scores. Tukey’s *post hoc* multiple comparison test was performed to identify pairwise comparisons between baseline and follow-up visits.

## 3 Results

### 3.1 Participant screening and enrolment

A total of 211 participants were screened for eligibility, and 104 eligible participants were enrolled in the study. Out of this, 61 participants dropped out of the study, and 43 participants successfully completed all the scheduled follow-ups. Among the 61 study dropouts, 25 participants did not return for further follow-up, while 36 participants attended less than 12 follow-up visits; however, the reason of study discontinuation could not be ascertained.

Among those who completed the study, 32 participants had Grade III and 11 had Grade IV lymphoedema. Out of these 43 participants, right leg was affected in 18 participants, left leg in 12 participants, and both legs in 13 participants. Overall, right leg was affected in 31 participants and left leg in 25 participants. The study flow diagram is illustrated in **Figure 1**.

**Figure 1:**
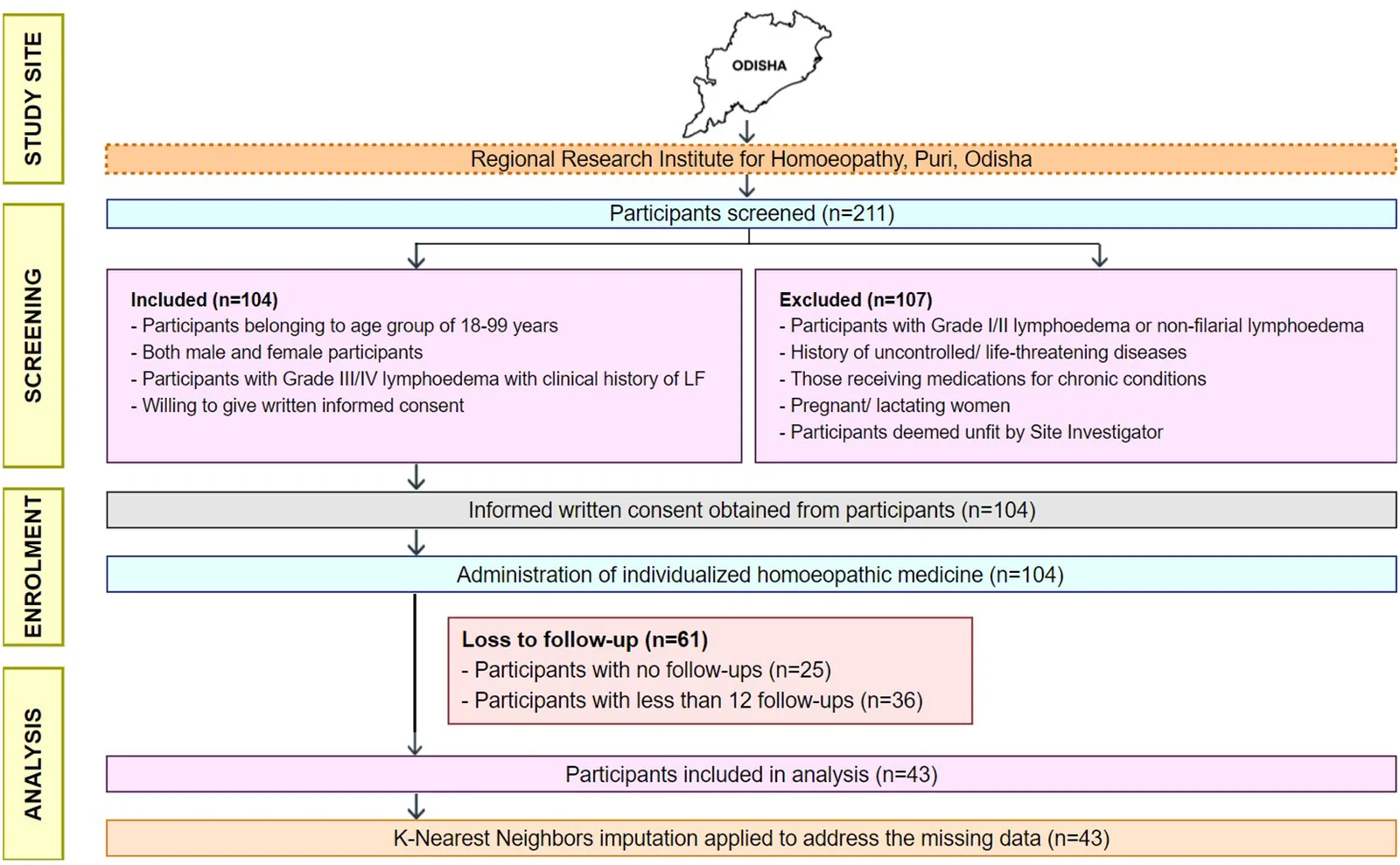
Study flow diagram.

### 3.2 Baseline demographic characteristics of study participants

The study population had a mean age of 55.56 ± 7.10, with a slight female preponderance (55.8%). The majority of the participants were married (90.7%), educated (58.1%), employed/self-employed (48.8%), and belonged to a low socio-economic status (83.7%). A family history of LF was reported in 55.8% of the participants. The baseline characteristics of the participants are represented in **Table 2**.

**Table 2:** Baseline characteristics of the study participants.

Variable	Grade III lymphoedema (n=32)	Grade IV lymphoedema (n=11)	Both grades (n=43)
Age (Mean ± SD)	54.50 ± 6.16	58.64 ± 8.95	55.56 ± 7.10
Gender [n (%)]			
Male	13 (40.6)	6 (54.5)	19 (44.2)
Female	19 (59.4)	5 (45.5)	24 (55.8)
Marital status [n (%)]			
Married	29 (90.6)	10 (90.9)	39 (90.7)
Unmarried/widowed	3 (9.4)	1 (9.1)	4 (9.3)
Education [n (%)]			
Educated	17 (53.1)	8 (72.7)	25 (58.1)
Uneducated	15 (46.9)	3 (27.3)	18 (41.9)
Occupation [n (%)]			
Employed/self-employed	13 (40.6)	8 (72.7)	21 (48.8)
Unemployed/retired	2 (6.3)	0 (0)	2 (4.7)
Homemaker	17 (53.1)	3 (27.3)	20 (46.5)
Socio-economic status [n (%)]			
High	1 (3.1)	0 (0)	1 (2.3)
Middle	4 (12.5)	2 (18.2)	6 (14.0)
Low	27 (84.4)	9 (81.8)	36 (83.7)
Family history of LF [n (%)]			
Present	19 (59.4)	5 (45.5)	24 (55.8)
Absent	13 (40.6)	6 (54.5)	19 (44.2)
Body mass index (Mean ± SD)	27.62 ± 5.84	25.35 ± 8.14	27.04 ± 6.48

n, Number of participants; SD, Standard Deviation; %, Percentage; LF, Lymphatic Filariasis.

### 3.3 Frequently prescribed medicines at baseline

The most frequently prescribed homoeopathic medicines at baseline were *Rhus toxicodendron* (n=8), *Lycopodium clavatum* (n=7), and *Phosphorus* (n=6), followed closely by *Natrium muriaticum* (n=5) and *Sulphur* (n=5) (**Table 3**).

**Table 3:** Frequently prescribed medicines at baseline.

S.No.	Name of medicine	Grade III lymphoedema (n=32)	Grade IV lymphoedema (n=11)
1.	*Rhus toxicodendron*	8	0
2.	*Lycopodium clavatum*	5	2
3.	*Phosphorus*	4	2
4.	*Natrium muriaticum*	3	2
5.	*Sulphur*	4	1
6.	*Silicea terra*	2	2
7.	*Arsenicum album*	2	0
8.	*Pulsatilla*	2	0
9.	*Acidum nitricum*	0	1
10.	*Calcarea carbonica*	1	0
11.	*Nux vomica*	0	1
12.	*Sepia officinalis*	1	0

n, Number of participants.

### 3.4 Change in limb circumference measurement

A statistically significant difference was observed in the absolute change in volume for the right leg (n=31) from baseline to 6^th^ and 9^th^ follow-ups (p-values 0.0008*** and 0.0131*, respectively). However, for the left leg (n=25), no statistically significant difference was observed in the absolute volume change (**Figures 2a, b**).

**Figure 2:**
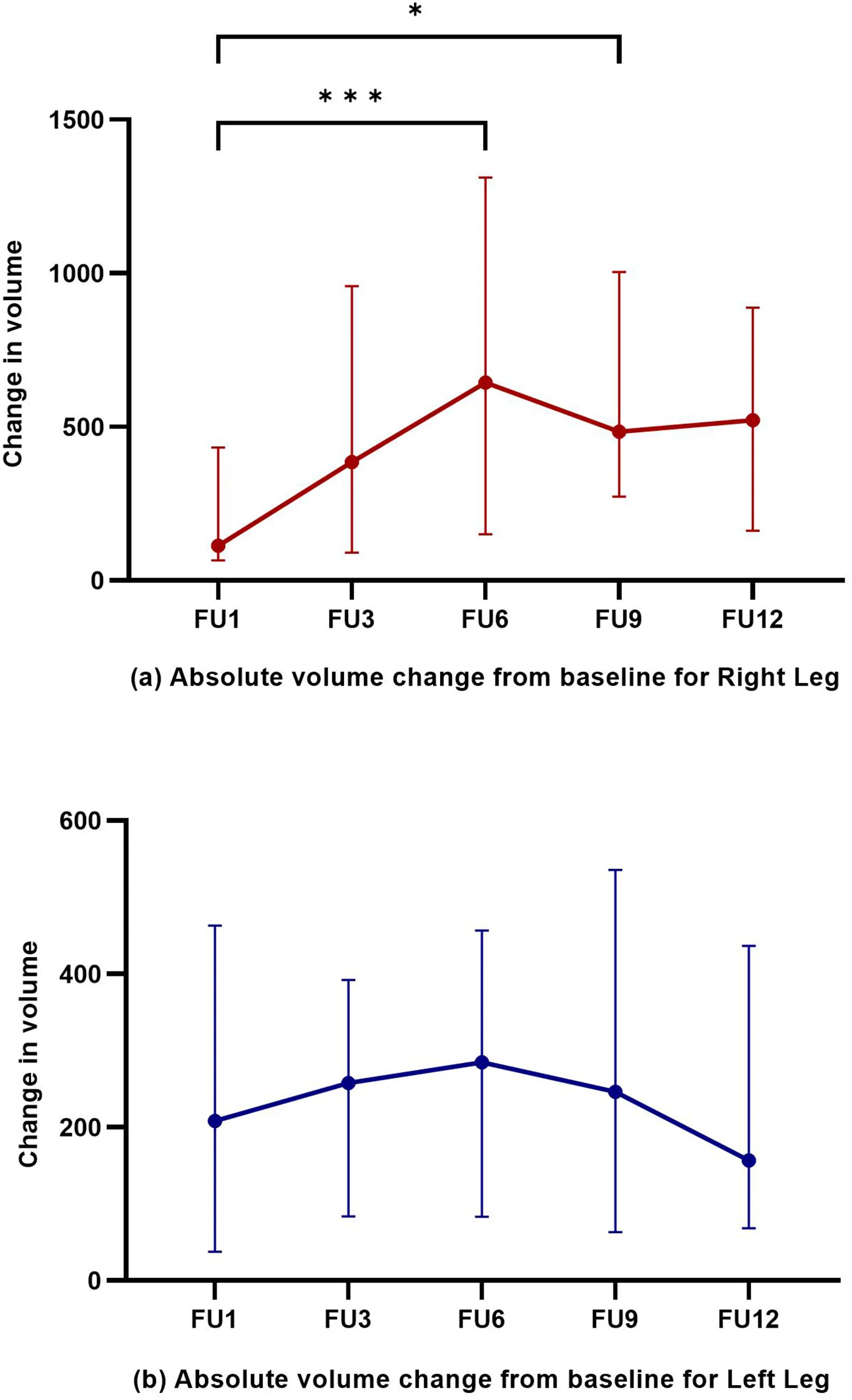
Absolute volume change in lower limb circumference measurement. **(a)** Absolute volume change from baseline for right leg. **(b)** Absolute volume change from baseline for left leg. * p-value < 0.5, *** p-value < 0.001.

### 3.5 Water volume change

A statistically significant change was observed in water volume for the right leg (n=31) between 1^st^ & 10^th^ follow-ups (p-value 0.0418*), 8^th^ & 10^th^ follow-ups (p-value 0.0117*), and 10^th^ & 12^th^ follow-ups (p-value 0.0397*). However, for the left leg (n=25), no statistically significant change was observed in the water volume (**Figures 3a, b**).

**Figure 3:**
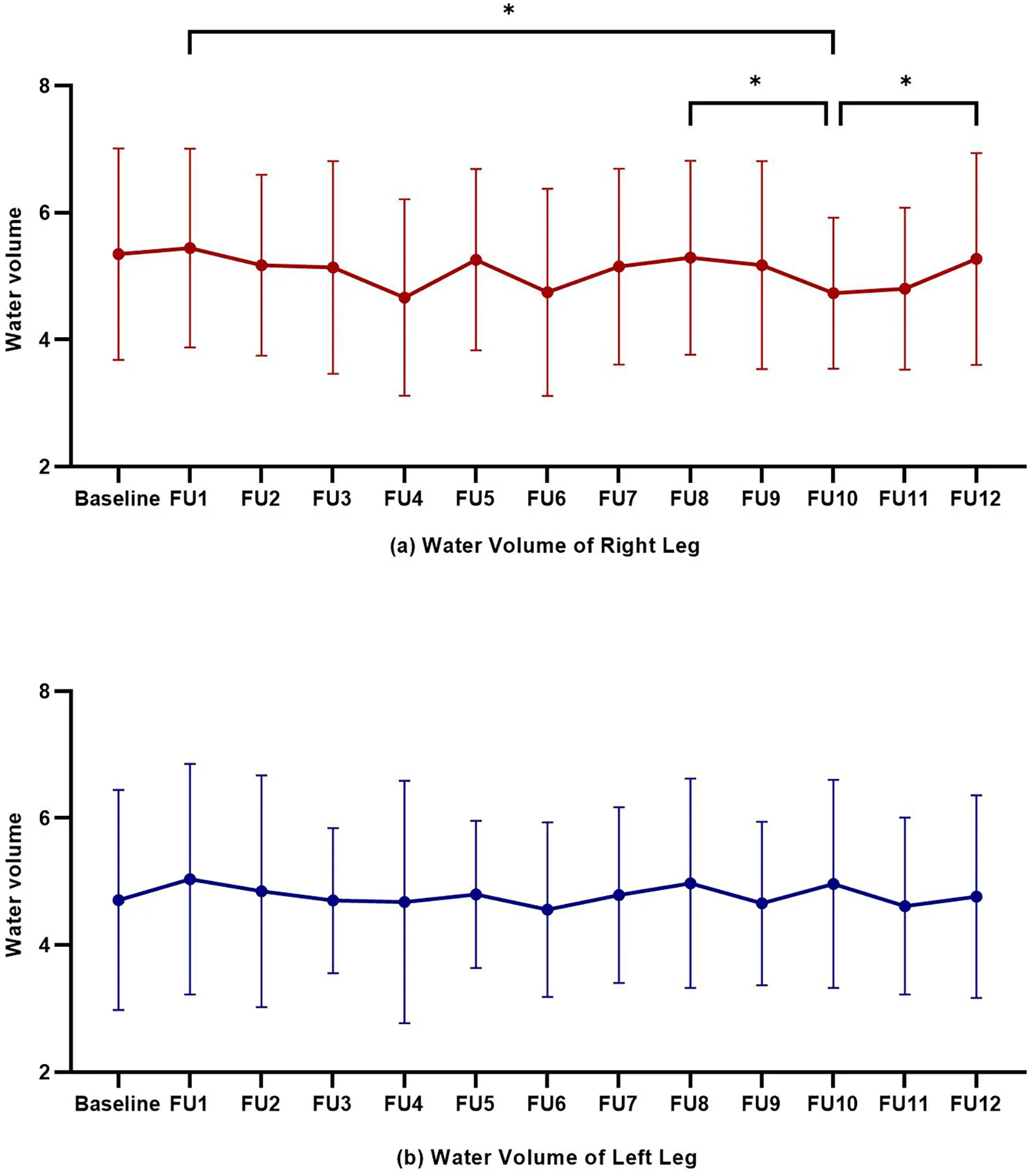
Change in water volume of right and left legs. **(a)** Water volume of right leg. **(b)** Water volume of left leg. * p-value < 0.05.

### 3.6 Change in 7D5L scores

A statistically significant reduction was observed in 7D5L scores from baseline to 6^th^, 9^th,^ and 12^th^ follow-up (p-values 0.0006***, <0.0001****, and 0.0003*** respectively). The mean score decreased from 14.16 ± 4.18 at baseline to 11.77 ± 3.74 after 12 months of intervention (**Figure 4**).

**Figure 4:**
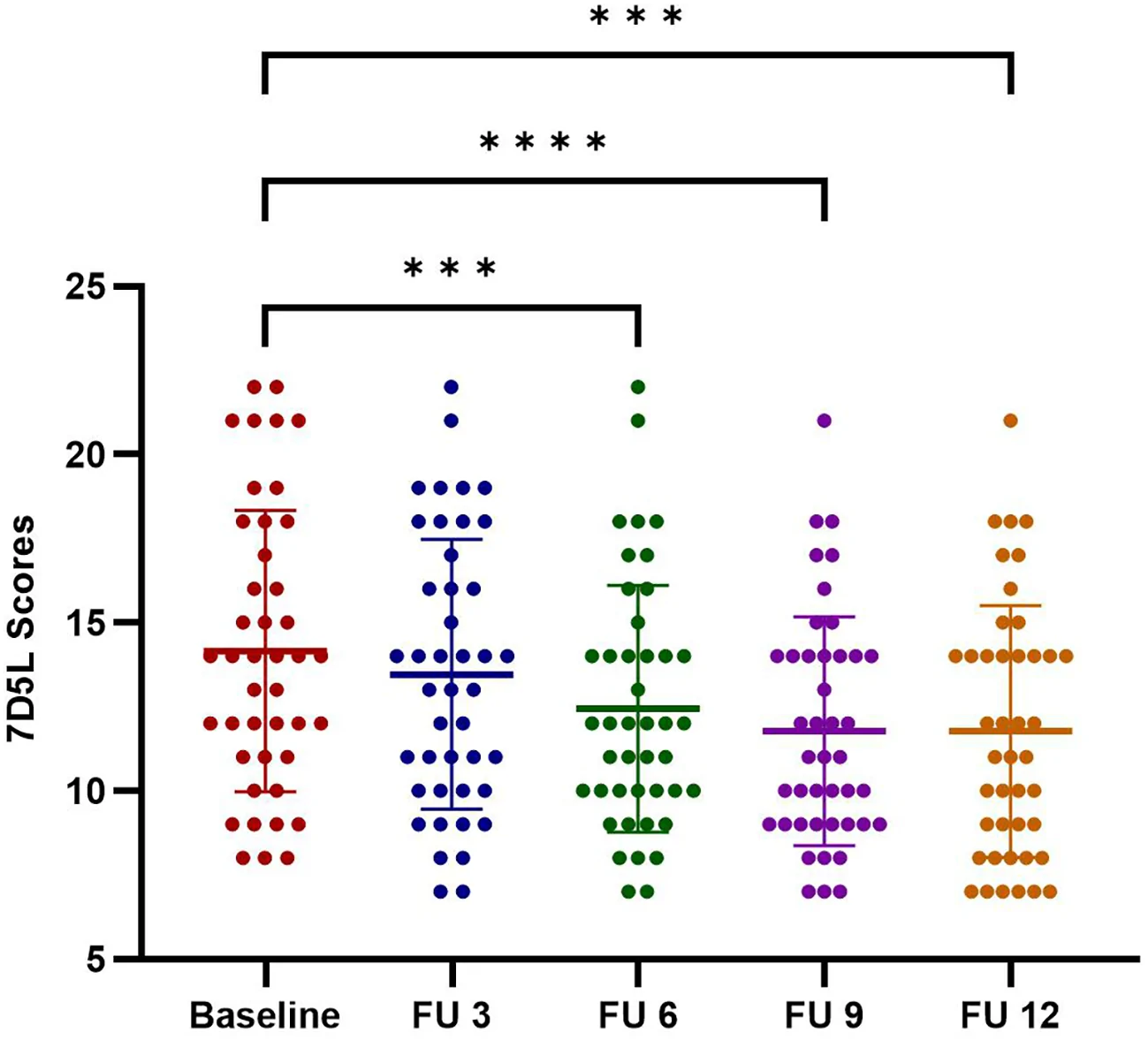
Change in 7D5L scores. *** p-value < 0.001, **** p-value < 0.0001.

Domain-wise analysis of 7D5L demonstrated statistically significant changes in scores over time. Significant improvements were observed from baseline in *mobility* at 12^th^ follow-up (p-value 0.0245*); *self-care* at 9^th^ and 12^th^ follow-ups (p-values 0.0263* and 0.0491*, respectively); and *usual activity* at 9^th^ follow-up (p-value 0.0437*). Additionally, *pain/discomfort* showed a significant difference from baseline at 6^th^, 9^th^ and 12^th^ follow-ups (p-values 0.0271*, 0.0015**, and 0.0004***, respectively); *anxiety/depression* at 3^rd^, 6^th^, 9^th^ and 12^th^ follow-ups (p-values 0.0077**, 0.0004***, <0.0001****, and 0.001***, respectively); *cognition* at 9^th^ follow-up (p-value 0.0043**); and *social participation* at 6^th^, 9^th^ and 12^th^ follow-ups (p-values 0.0458*, 0.0088**, and 0.0400* respectively).

### 3.7 Change in DLQI scores

A statistically significant reduction was observed in DLQI scores from baseline to 6^th^, 9^th,^ and 12^th^ follow-ups (p-value < 0.0001****). The mean DLQI score decreased from 9.79 ± 5.39 at baseline to 5.63 ± 4.49 at 12^th^ follow-up (**Figure 5**).

**Figure 5:**
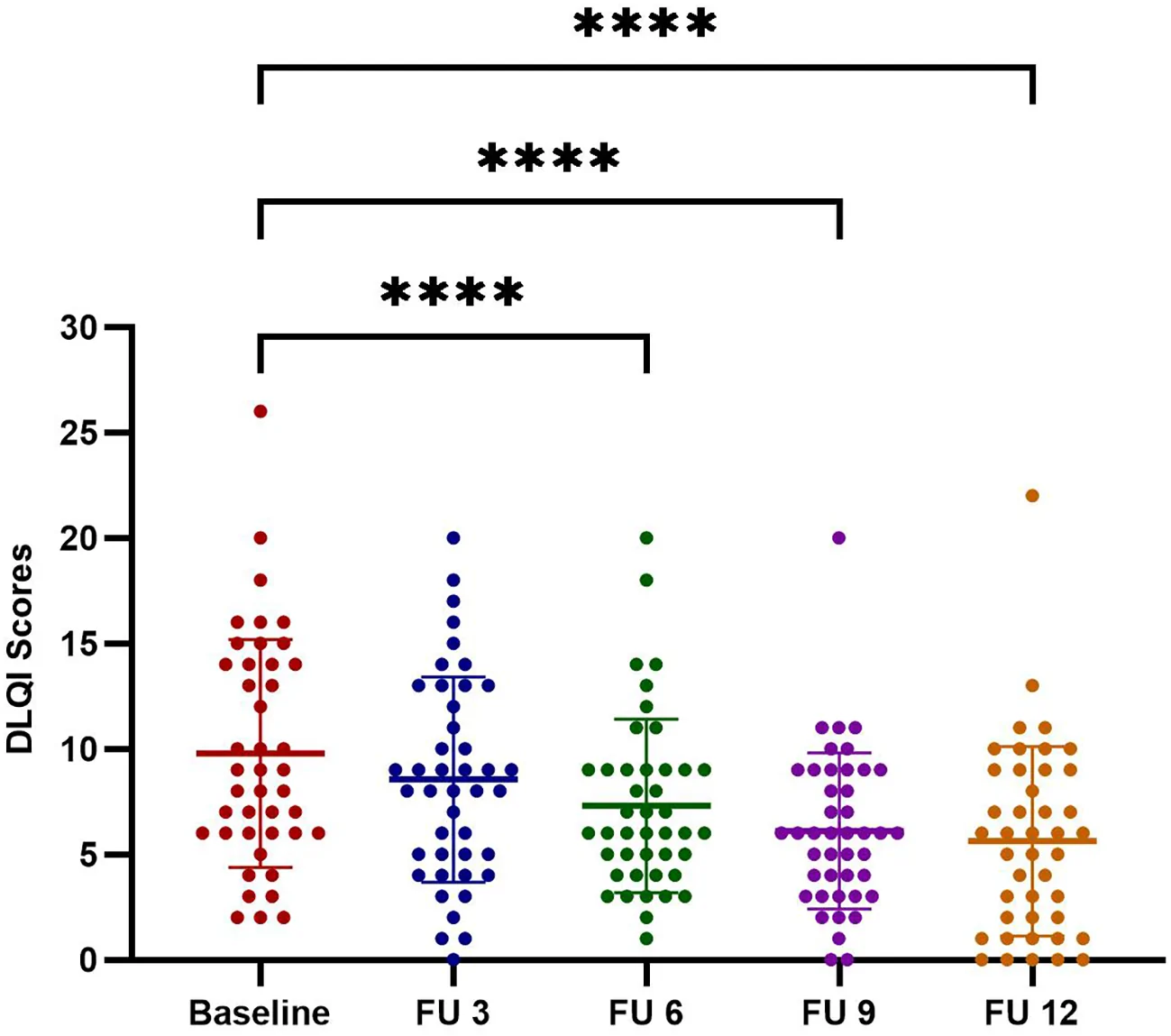
Change in DLQI scores. **** p-value < 0.0001.

### 3.8 Serological estimation of Og4C3 and TNF-α

The mean Og4C3 value reduced from 2.26 ± 9.11 at baseline to 0.32 ± 1.16 at 12^th^ follow-up. However, this change was not statistically significant (p-value 0.2675). Similarly, the mean *TNF-α value decreased from 8.14* ± *29.19* at baseline to 3.49 ± 11.80 at 12^th^ follow-up; however, no statistically significant change was observed (p-value 0.6387).

### 3.9 Number of acute adenolymphangitis attacks

The number of acute adenolymphangitis attacks in past six months was assessed based on participant self-report at baseline. Among the 43 participants, 20 (46.5%) reported no acute adenolymphangitis episodes, while 23 (53.5%) reported atleast one episode at baseline. However, 21 (48.8%) participants reported no acute adenolymphangitis episodes, and 22 (51.2%) reported at least one episode at the 12^th^ follow-up.

### 3.10 Skin health with respect to entry lesions, fungal infection, and ulcer complications

Only one participant presented with entry lesions and ulcer complications at baseline, which subsided after 3 months of treatment (**Figures 6a, c**).

**Figure 6:**
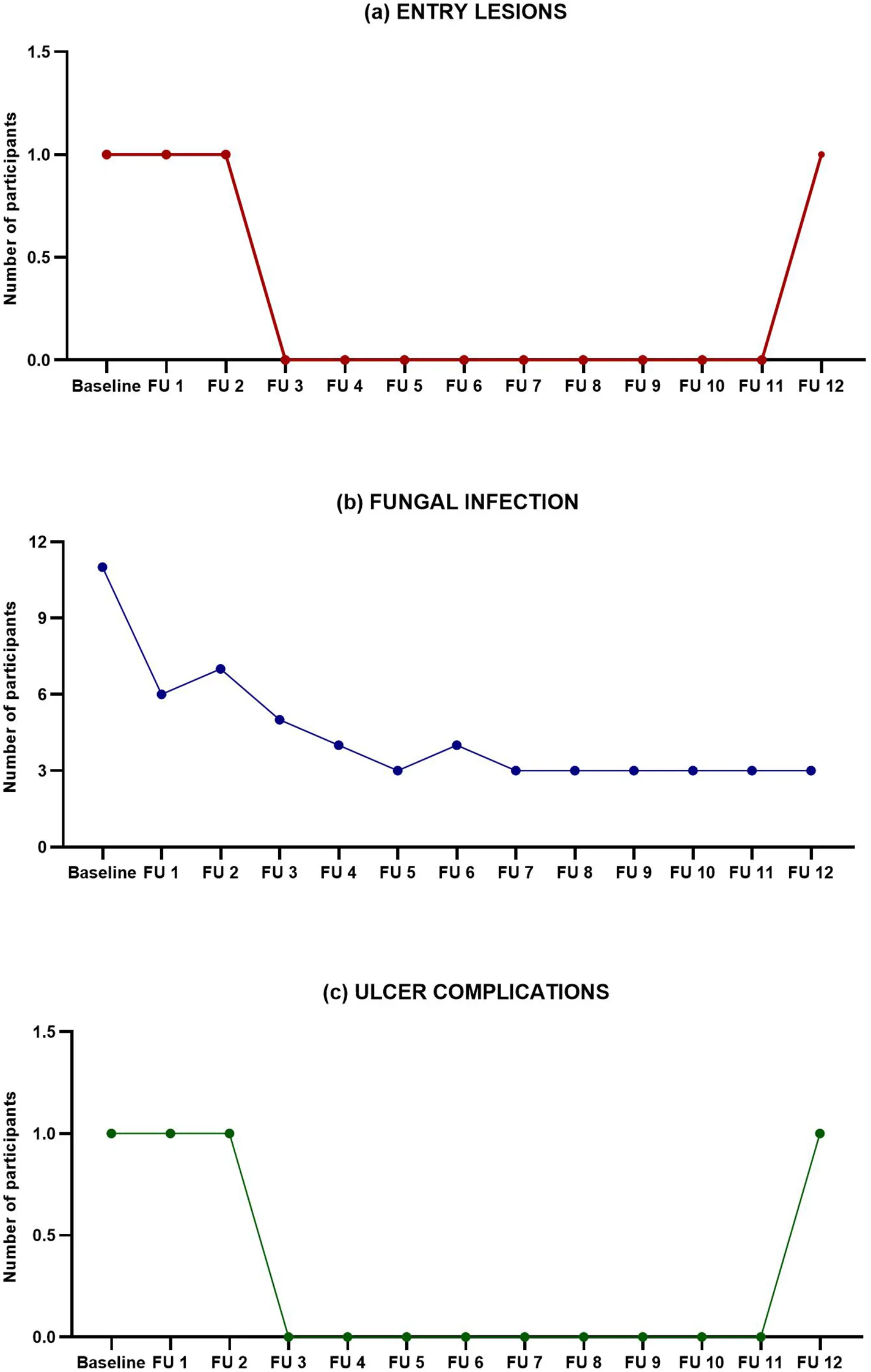
Trends in skin health related complications during follow-ups. **(a)** Entry lesions **(b)** Fungal Infection **(c)** Ulcer complications.

11 participants had fungal infections at baseline; however, only 03 participants had fungal infections at the 12^th^ follow-up (**Figure 6b**).

## 4 Discussion

This non-randomized interventional study demonstrated the probable therapeutic effects of individualized homoeopathic medicines in managing LF-associated advanced (grade III and IV) lymphoedema. Earlier, a multicentric trial conducted by CCRH suggested the role of homoeopathy in managing early-stage lymphoedema (grade I and II) (Prusty et al., 2025). Together, these studies have expanded the horizon, highlighting the scope of homoeopathy in addressing the diverse needs of patients with lymphoedema, from early-stage management to more complex and advanced cases.

The study population had a slight female preponderance (55.8%) and predominantly belonged to low socio-economic status (83.7%). Notably, a significant proportion (55.8%) reported a family history of LF. These findings are consistent with previous research studies, which highlight the disproportionate burden of LF on vulnerable populations, particularly women and those from low socio-economic backgrounds. For instance, a study conducted in a rural coastal Odisha showed that acute adenolymphangitis attacks resulted in significant economic burden and productivity loss, especially among females. In this regard, Homeopathy may offer a cost-effective approach to mitigate the impact of LF (Babu and Nayak, 2003).

The significant reduction in limb volume observed in the right leg, coupled with improvements in health-related quality of life and dermatological symptoms, suggests the potential clinical relevance of homoeopathy as an adjunctive approach in the management of morbidity associated with advanced lymphoedema. In the present study, the statistically significant changes in 7D5L scores were noted, particularly in patient-reported domains such as pain/discomfort, and anxiety/depression, which indicate a notable improvement in the overall quality of life of participants. The findings are clinically relevant because advanced lymphoedema can substantially affect physical functioning, psychological well-being, and participation in daily and social activities. These observed improvements align with previous studies, which found that homoeopathic medicines, when administered early, prevented the worsening of lymphoedema and led to enhanced overall well-being among participants (Mishra, 1998; Prusty et al., 2025).

The dermatological symptoms also showed marked improvement, as evidenced by the significant reduction in DLQI scores. The improvement in skin health, including the resolution of entry lesions and ulcer complications, and the reduction in fungal infections, may be clinically relevant because preservation of skin integrity is crucial to managing morbidity in chronic lymphoedema. These findings are consistent with the study, which found that homoeopathic medicines may help manage active disease phases (Rastogi and Mishra, 1991; Prusty et al., 2025). However, these observations may be regarded as descriptive and hypothesis-generating rather than evidence of a definitive treatment effect, since the number of participants with entry lesions, ulcer complications, and fungal infections was less.

Acute adenolymphangitis is a significant contributor to morbidity in lymphatic filariasis and may worsen physical functioning and quality of life. In this study, the proportion of participants reporting no acute attacks showed only a modest change from baseline to the 12-month assessment. As a result, there was no discernible decrease in the frequency of acute adenolymphangitis attacks over the study period. Hence, this outcome should be interpreted in conjunction with the noted improvements in quality of life, dermatological health, and limb volume rather than being regarded as evidence of a specific impact on acute inflammatory episodes.

Various studies have reported that the LF-affected individuals exhibit increased levels of inflammatory markers, including C-reactive protein and cytokines such as TNF-α, IL-2, IL-6, IL-8, etc. (Babu and Nutman, 2014) Although the changes in Og4C3 and TNF-α values were not statistically significant in this study, the trend towards reduction suggested a possible anti-inflammatory effect of homoeopathic medicines. Further studies with a larger sample size and a rigorous study design might prove beneficial to confirm these findings.

The most frequently prescribed homoeopathic medicines in this study were *Rhus toxicodendron*, *Lycopodium clavatum*, *Phosphorus*, *Natrium muriaticum*, and *Sulphur*. A study by Gupta et al. also reported *Rhus toxicodendron* as one of the frequently prescribed medicines for acute attacks (Gupta et al., 2018). The conventional MDA therapies including diethylcarbamazine, ivermectin, and albendazole, are known to modulate molecular pathways involved in microfilarial clearance; however, their macrofilaricidal action remains limited. Pertinently, these therapies may not be effective in managing advanced grade III and IV lymphatic filariasis, where lymphatic obstruction caused by adult worms predominates (Centers for Disease Control and Prevention, 2024). In this regard, the action of homoeopathic medicines used in this study may be hypothesized to stabilize or potentially reverse disease progression by modulating the host immune responses. Future studies are required to explore the mechanisms underlying the reduction in limb swelling observed in this study, despite the advancement in lymphoedema, which may open new avenues for pharmacological research in LF.

Adverse events are commonly reported following the LF treatment, and concerns regarding such events often reduce compliance with MDA programmes (Laman et al., 2022). Notably, no adverse or serious adverse events were reported during any follow-up visit in the present study, suggesting that the homoeopathic interventions were well tolerated by the participants.

The findings of this study have significant implications for the management of lymphoedema in resource-poor settings, particularly in LF-endemic regions, where the disease burden is high and access to specialized care is limited. Homoeopathy, being a relatively low-cost and accessible intervention, may offer a valuable adjunct to existing treatment modalities for patients with advanced lymphoedema. By integrating Homoeopathy into existing healthcare settings and national filaria elimination strategies, it may also be possible to improve health outcomes and reduce the economic burden of LF in endemic regions.

While the study findings are promising, several limitations were observed. The study had a small sample size, the absence of a comparator arm, and a lack of randomization or blinding, collectively limiting the internal validity and generalizability of the findings. In addition, the open-label design may have introduced confirmation and instrument bias, while the subjective components of outcome assessment could be influenced by observer bias.

The individualized nature of the intervention and the response-dependent repetition of medicines may have introduced variability in the treatment exposure across participants. Since the potency, dose, and frequency of the medicine were determined based on individual clinical response, a standardized treatment schedule could not be maintained, potentially affecting the reproducibility of the intervention and the interpretation of outcomes relative to a specific treatment regimen.

Although the study included the biochemical evaluation using Og4C3 and TNF-α serological markers, the complex immunological and inflammatory pathways involved in LF were not explored. The absence of a broader panel of inflammatory markers restricted a more comprehensive understanding of the underlying biological effects. The study relied on clinical and laboratory assessments without advanced imaging or functional lymphatic evaluations, which may have limited the precision of disease-severity assessment and the objective measurement of treatment response. Furthermore, the use of multiple outcome measures, including quality of life and dermatological indices, while comprehensive, may also increase the risk of measurement variability, particularly in the absence of centralized or blinded assessment.

Potential confounding factors, particularly concurrent supportive care, limb hygiene practices, nutrition, and environmental influences, were not fully controlled or systematically standardized across participants. These measures were not provided as protocol-specific interventions, although participants may have independently practiced them during the study period. Such factors may have influenced some clinical and dermatological outcomes. Therefore, the findings should be interpreted in the context of the overall intervention, while acknowledging that the contribution of unrecorded concomitant care cannot be excluded. Future controlled studies should prospectively document and standardize concomitant lymphoedema care to better delineate the independent contribution of the homoeopathic intervention.

The sample size estimation was based on a single-proportion approach because of limited prior evidence regarding the expected improvement in morbidity indicators. As the study employed repeated-measures analyses across multiple follow-up time points, this approach did not directly account for within-subject correlations or the expected effect size for longitudinal changes. This represents a methodological limitation in the sample size estimate and should be considered when interpreting the study’s findings. Future controlled studies with prospective documentation of concomitant care and longitudinal sample-size estimation based on expected effect sizes would help to better delineate the independent contribution of the homoeopathic intervention.

The COVID-19 pandemic prevalent during the study period posed challenges to participant recruitment, follow-up, and retention, necessitating an extension of the study period to achieve the pre-determined sample size. Despite the study extension, the target sample size could not be achieved due to a substantial dropout rate. As many participants travelled to the study site from neighbouring districts, restrictions on transportation and movement during the pandemic may have contributed to difficulties in attending scheduled follow-up visits. In addition, the study did not have provisions for telephonic follow-up or home visits, which may have further limited the ability to maintain follow-up among participants who were unable to attend the study site. However, the specific reasons for study discontinuation could not be ascertained. A formal baseline comparison between study completers and non-completers could not be performed reliably because complete baseline data were unavailable for all participants who discontinued the study. Hence, potential baseline differences between these groups and their possible contribution to attrition bias cannot be excluded, which may have potentially affected the internal validity and generalizability of the findings.

For participants who completed the study and were included in the longitudinal analysis, KNN imputation was applied to address missing data across follow-up assessments; however, this approach may not completely eliminate the uncertainty associated with missing data and does not address potential bias arising from non-random dropout.

Further, the study protocol was not published prior to the study initiation. However, reporting was done adhering to the STROBE guidelines (von Elm et al., 2008) ensuring comprehensive coverage of key items.

## 5 Conclusion

The study demonstrated the potential benefits of Homoeopathy in managing grade III and IV lymphoedema associated with LF, particularly in improving health-related quality of life, reducing dermatological symptoms, and alleviating limb volume. The observed improvements in patient-reported domains, including mobility, self-care, pain/discomfort, psychological health and social participation, suggest that the potential clinical relevance of the intervention may extend beyond changes in limb measurements to aspects of daily functioning and well-being. Although the findings are encouraging, they should be considered exploratory and hypothesis-generating rather than confirmatory. These observations support further investigation of the potential role of Homoeopathy as an adjunct in morbidity management and disability prevention among individuals affected with LF, including its plausible inclusion within MMDP activities. Further validation through larger multicentric randomized controlled trials incorporating appropriate comparator groups, and broader biomarker profiling is warranted to assess the efficacy, reproducibility, causal attribution and long-term sustainability of homoeopathic interventions.

From a public health perspective, achieving sustainable LF elimination will require a comprehensive One Health framework that integrates public health, environmental, zoonotic, and vector-control surveillance to address persistent transmission dynamics. Strengthening advanced diagnostic capabilities alongside community-based awareness initiatives may facilitate early detection and timely intervention. Moreover, sustained multisectoral coordination and strategic policy support, particularly through long-term resource allocation for mass drug administration, morbidity management and disability prevention programs, and digital health infrastructure, will remain pivotal to advancing resilient and sustainable LF control and elimination efforts.

## Data Availability

The original contributions presented in the study are included in the article. Further inquiries can be directed to the corresponding author.
